# Effects of Anserine/Carnosine Supplementation on Mild Cognitive Impairment with APOE4

**DOI:** 10.3390/nu11071626

**Published:** 2019-07-17

**Authors:** Nobutaka Masuoka, Chitose Yoshimine, Marie Hori, Mieko Tanaka, Takashi Asada, Keiichi Abe, Tatsuhiro Hisatsune

**Affiliations:** 1 Department of Integrated Biosciences edu., Graduate School of Frontier Sciences, The University of Tokyo, Kashiwa 277-8562, Japan; 2 Brain Functions Laboratory, Inc., Yokohama 230-0046, Japan; 3 Memory Clinic Ochanomizu, Bunkyo-ku, Tokyo 113-0034, Japan; 4 Center for Brain Integration Research, Tokyo Medical and Dental University, Bunkyo-ku, Tokyo 113-8519, Japan; 5 National Institute of Health and Nutrition, National Institute of Biomedical Innovation, Health and Nutrition, Shinjuku -ku, Tokyo 162-8636, Japan

**Keywords:** Alzheimer’s disease, cognitive functions, anserine and carnosine, randomized controlled trial (RCT), mild cognitive impairment (MCI), APOE4

## Abstract

Background: Oral supplementation of anserine/carnosine helps preserve cognitive functions in healthy older adults. Mild cognitive impairment (MCI) is a transition between cognitive-normal and dementia. Therefore, it needs to investigate whether anserine/carnosine supplementation (ACS) has effects on subjects with MCI. Methods: A randomized, double-blind, placebo-controlled 12-week trial was performed. Fifty-four subjects with MCI were randomized to an active group ingesting 750 mg of anserine and 250 mg of carnosine per day or a placebo (1:1). Evaluation of cognitive change was conducted utilizing a psychometric test battery. Results: The score improvement in the global Clinical Dementia Rating (gloCDR) was superior in the active group than placebo (*p* = 0.023). No beneficial effect in the active group was detected in the other psychometric tests including the Mini-Mental State Examination (MMSE), the Wechsler Memory Scale, and the Alzheimer’s Disease Assessment Scale (ADAS). When APOE4 positive (APOE4 (+)) or negative (APOE4 (-)) subjects were separately analyzed, beneficial change in the APOE4 (+) subjects was observed in MMSE (*p* = 0.025) as well as in gloCDR (*p* = 0.026). Conclusions: The present study might suggest that protective effects against cognitive decline in APOE4 (+) MCI subjects exist.

## 1. Introduction

Fifty million people worldwide have dementia [1,2]. In individuals aged more than 70 years, dementia is the second largest cause of death [3]. Alzheimer’s disease (AD) is the most commonly observed form of dementia that composes nearly 70% of cases [4]. The most influential genetic risk factor known for AD is the inheritance of the ε4 allele of apolipoprotein E (APOE4) [5,6,7,8]. The sign of AD typically starts with memory decline. On the other hand, Mild Cognitive Impairment (MCI) is diagnosed when there is evidence of memory impairment, but the patient retains general cognitive function and activities of daily living and is not demented [9]. MCI is sometimes a transition between aging and dementia [10]. It is reported that more than 10% of MCI individuals develop dementia in a year [11]. Therefore, beneficial intervention on MCI should be sought in order to decrease the number of patients who suffer from AD in the future. Current evidence does not support the use of pharmacologic treatments for cognitive protection in persons with MCI [12]. Thus, intervention to MCI aiming to reduce risk from AD dementia onset could target lifestyle improvements including augmentation of intellectual or physical activities, and nutritional adjustment [13,14,15].

In randomized controlled trials from our laboratory and from others, anserine and carnosine supplementation (ACS) in seniors helped to preserve cognitive function, primarily verbal episodic memory, and brain perfusion [16,17,18,19]. Carnosine is an anti-inflammatory dipeptide, consisting of beta-alanine and histidine, and is present in the millimolar range in skeletal muscle, and in the hundred-micromolar range in the vertebrate brain [20]. Anserine is a natural derivative of carnosine and is present at high levels in the breast skeletal muscle in chicken. Anserine and carnosine are called as imidazole dipeptides, which have physiological functions in common such as anti-oxidation, pH buffering, metallic chelation, but anserine is not cleaved by human carnosinase, which is abundant in human serum [21,22]. Beneficial effects of ACS, which was observed in the cognition of healthy elderly subjects, led us to investigate the effects of ACS in MCI individuals with hope for finding any benefit for dementia prevention. Moreover, the results of our previous trial showed that ACS preserved brain perfusion in individuals carrying APOE4 [19]. However, efforts for almost 25 years to elucidate the mechanism of how this allele increases the risk for AD onset have not yielded enough results.

In the present study, we conducted a randomized, double-blind, placebo-controlled trial of oral ACS for 12 weeks with subjects of MCI patients referring to a memory clinic in the Tokyo Metro Area. APOE4 genotyping of participants was performed, then the results were utilized in the subanalysis conducted separately for the subjects with or without APOE4. MCI patients were enrolled in the trial and randomized to enter the active group (Active group) or the placebo group (Placebo group). They consumed 750 mg of anserine and 250 mg of carnosine per day or a placebo. The dose of anserine and carnosine for supplementation was equivalent to that in previous studies. [16,17,18,19]. At the baseline and follow-up, psychometric examinations including MMSE and Clinical Dementia Rating (CDR) were performed. Participants also underwent electroencephalograms (EEG) recording, and the data were used to estimate cortical neural activity impairment that is sensitive to the early stage of AD. [23,24,25]. This study aimed to evaluate the effects of ACS on cognitive decline in MCI individuals, and as secondary, to consider the capability of ACS to alleviate cognitive deterioration by APOE4.

## 2. Materials and Methods

### 2.1. Study Design and Participants

This study was a randomized, double-blind, placebo-controlled trial randomizing 1:1 active: Placebo conducted to evaluate the efficacy of ACS on cognitive function in MCI individuals. This study was approved by the Ethics Committee of The University of Tokyo (ID: 15-117, 14 September 2015). This study was registered in the UMIN Clinical Trials Registry (ID: UMIN R000023455), and carried out following the Declaration of Helsinki and the Ethical Guidelines for Medical and Health Research Involving Human Subjects. Participants were volunteers among outpatients referring to a memory clinic located in the Tokyo Metro Area from January to November in 2016. The Clinical Dementia Rating (CDR) was utilized to adopt subjects from outpatients. The global Clinical Dementia Rating (gloCDR) score of all participant candidates was 0.5 at the baseline [26]. A responsible doctor gave information on this study to the candidates, confirming their clinical assessment was within the Mild Neurocognitive Disorder in DSM-5 [27]. Participants provided written informed consent. Exclusion criteria were as follows: (1) The score of Mini-Mental State Examination (MMSE) was 23 or less, (2) the score of the Geriatric Depression Scale-Short Version–Japanese (GDS-S-J) was six or above, (3) the usage of donepezil, galantamine, rivastigmine, or memantine was changed in the previous six months [28], (4) a patient had a history of a neuropsychiatric disorder, a head injury, or a local brain lesion which could affect cognition such as tumor or cerebral infarction, (5) a patient was allergic to chicken, because the test supplement was derived from [16,17], (6) a responsible doctor considered that a patient was inappropriate for participation.

**Figure 1:**
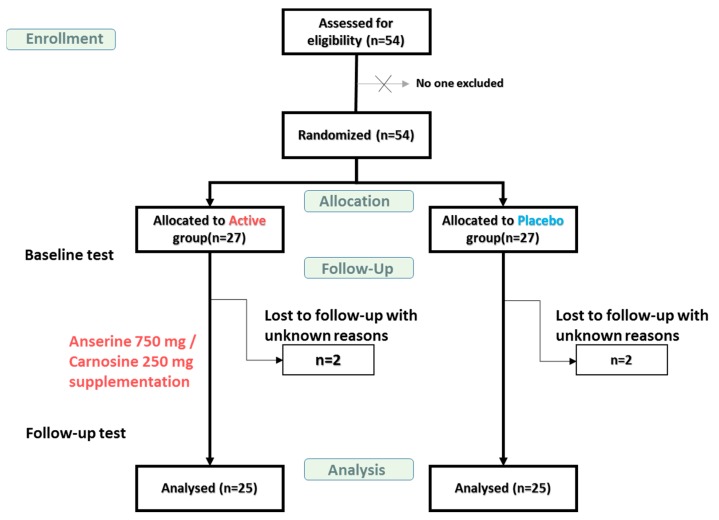
Flow diagram showing the number of mild cognitive impairment (MCI) participants during the study. Baseline test: At the beginning of the test. Follow-up test: Twelve weeks after beginning.

This study was designed to detect the 0.1 points difference for the gloCDR sore at follow-up assuming a standard deviation (SD) of 0.18 with a type 1 error protection of 0.05 two-sided and 80% of the power. The number of subjects needed was calculated to be 52. We conducted the present study with 54 participants aged 49 to 86 years (average; 72.8 years). They were randomized to enter the Active or Placebo group in ratio 1:1 as Figure 1. The actual allocation of participants into either group was done matching up the age and the sex ratio in the two groups by Imepro Inc. (Tokyo, Japan). Participants underwent first examinations at the beginning of the trial and were given a supply of either ACS or a placebo for consumption at home, after which they returned to the clinic every four weeks for a new supply, and then finally underwent tests at follow-up in 12 weeks. Clinical staffs, coordinators, and participants were blinded to the group assignments for the duration of the trial. Of 54 participants, the total of 50 consisting of 25 each in the ACS and Placebo group completed the 12-week trial and the tests at follow-up, and the data from the 50 subjects were included in the statistical analysis.

### 2.2. Inventory of Anserine and Carnosine in the Normal Diet

A dietary survey was conducted using a semi-quantitative method reported elsewhere to estimate anserine and carnosine intake from the usual diet [17,29]. At the baseline and follow-up, participants filled out a self-administered questionnaire on the frequency of animal meat (chicken, pork, and beef) or fish (divided into salmon, red-meat fish represented by tuna, white fish, blue-back fish represented by mackerel, and eel) in their diet during the previous 12 weeks. The representative fish in each category was based on a national consumption survey. Average concentrations of anserine and carnosine in these food materials were obtained from our preliminary examinations and from Boldyrev et al. [20]. The average concentration and response in the questionnaire were combined to calculate the estimated intake of anserine and carnosine in the subjects’ usual diet.

### 2.3. Blood Analysis, APOE4 Genotyping and ELISA for Amyloid Beta 42

Blood samples of the participants were collected at the baseline and follow-up. The samples were delivered to Kotobiken Medical Laboratories, Inc. (Tokyo, Japan) for blood count and biochemistry. APOE4 genotyping was conducted with blood samples [30], based on the informed consent provided by the participants. The plasma from APOE4 (+) subjects was heparinized and provided for the measurement of Amyloid beta protein (Aβ) 1-42. The measurement was done in our laboratory with the human/rat Aβ (1-42) ELISA kit (FUJIFILM Wako Pure Chemical Corporation) using the heparinized samples diluted with the same volume of saline.

### 2.4. Test Formulae

Test formulae were powder derived from chicken, which contained anserine and carnosine with an approximate ratio of 3:1 by weight. Creatine and creatinine were removed from the primary material by a two-step chromatography by Tokai Bussan Co., Ltd. (Tokyo, Japan) before use in the trial. Participants in the Active group received 750 mg of anserine and 250 mg of carnosine per day in 12 capsules to ingest six capsules twice a day, while participants in the Placebo group ingested a placebo, which is free of imidazole dipeptides in the same number of capsules, for 12 weeks. 

### 2.5. Cognitive Testing

The cognitive function on MCI participants in the trial was evaluated by psychometric examinations including MMSE, the Clinical Dementia Rating (CDR), the Alzheimer’s Disease Assessment Scale (ADAS), the Wechsler Memory Scale-Revised Logical Memory Immediate Recall (WMS-1) and Delayed Recall (WMS-2), and by clinical assessment by medical specialists. Higher scores mean better cognitive performances in MMSE, WMS-1, and WMS-2, while lower scores do in CDR and ADAS. Individuals with a gloCDR score of 1.0 were often at the level of cognitive functions in the dementia onset. The Geriatric Depression Scale (GDS) was used to evaluate a mood state [16,17,30,31]. 

### 2.6. EEG Recording

Electroencephalograms (EEG) were recorded with the patient awake and in resting, with eyes closed for 5 min before the test. Scalp potentials were recorded with 21 electrodes located at Fp1, Fp2, F3, F4, F7, F8, Fpz, Fz, T3, T4, T5, T6, C3, C4, Cz, P3, P4, Pz, O1, O2, and Oz of the International 10–20 System, as previously described [23]. EEG data were sampled at 200 Hz per channel and band-pass filtered to pick up signals at a frequency of 4–20 Hz. The recorded signal sequence was divided into segments of 0.64 s, which we previously found to be optimal for data analysis [23]. 

### 2.7. Adverse Events and Safety

The office of the study controller and the clinic received phone calls to hear any adverse events throughout the study. Medical specialists who saw participants every four weeks at the clinic were ready to consider the cause-and-effect relationship in adverse events during the trial, if any, and decided the possibility of continuance for each participant.

### 2.8. Statistical Analysis

To examine the effects of ACS on cognitive functions, we performed a two-way analysis of Treatment × Time Interaction. A *p* value of less than 0.05 was defined as statistically significant. The items used as covariates were age, gender, body mass index, and years of education. A multivariate analysis tool in the Microsoft Excel add-in program was used. To examine the effects of ACS on the concentration of *Aβ1-42* in plasma, we performed a two-way analysis of Treatment × Time Interaction. To analyze the correlation between the MMSE score and the value from the EEG analysis, we performed the Pearson correlation analysis.

## 3. Results

### 3.1. Characteristics of the Subjects

Data were analyzed from 50 subjects with MCI who were randomly assigned to the Active and Placebo groups (25 per group) and who completed the 12-week study, including the baseline and follow-up tests (Figure 1). Table 1 shows the subject characteristics. The Active and Placebo groups did not differ significantly concerning age, body mass index, or education, and the ratio of sexes in each group was identical. The proportion of APOE4 carriers among the 50 subjects in the study was 40.0%. One subject in the Active group and four in the Placebo group were APOE4-homozygous (APOE4/4 subjects). The results of the complete blood count and blood biochemistry are shown in Table S1.

### 3.2. Anserine and Carnosine Intake from the Usual Diet in the Study Population

Normal diets contain some anserine and carnosine. The subjects’ estimated dietary intake of anserine from the meat of domesticated animals and fish during the trial was calculated from an 8-item dietary questionnaire about the frequency of eating meat or fish and the type eaten, as described previously [17,29]. There was no significant difference between the groups in the estimated daily dietary intake for anserine, which was 306 mg for the Active and 347 mg for the Placebo group, or for carnosine, which was 178 mg for the Active and 183 mg for the Placebo group (Table S2). The daily ACS was 750 mg anserine and 250 mg carnosine. Thus, the Active group received approximately three times more anserine/carnosine than the Placebo group in this trial. An estimated intake of anserine and carnosine was calculated from the results of eight-item food frequency questionnaires filled out by each volunteer and the average amount of anserine and carnosine in each type of meat described by Boldyrev et al. [20].

### 3.3. Primary Analysis

**Figure 2:**
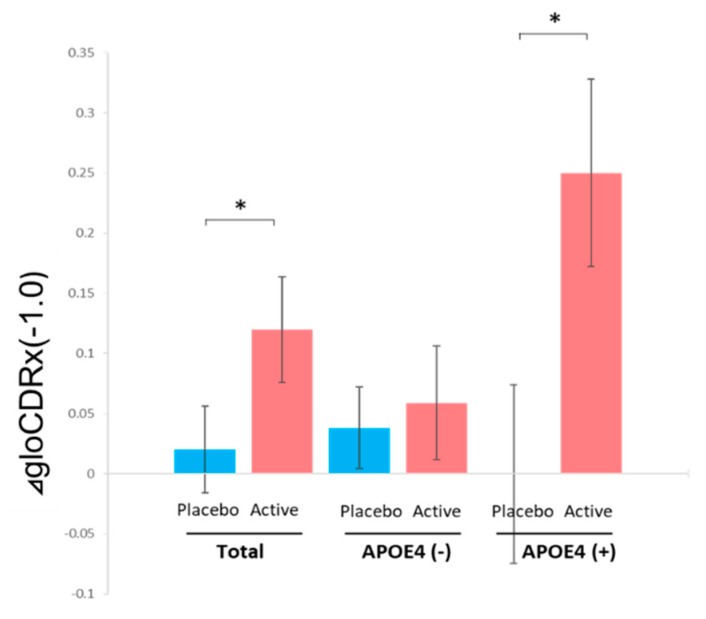
The comparison of the global (glo)CDR score improvement between the placebo-administered subjects and the anserine/carnosine supplementation (ACS)-administered subjects for the total or APOE4 negative/positive participants. A bar shows the average of the data and ± SEM. * represents *p* < 0.05 as described in Table 2 and Table 3.

No significant difference was observed in the changes of cognitive tests performed; MMSE, WMS-1, WMS-2 or ADAS, or in the change of GDS between the Active and Placebo group during the trial. In CDR, the score of the CDR sum of boxes (CDRsob) did not change in either of the two groups and the changes of the scores in each group were not statistically different, while the global CDR (gloCDR) after the Treatment × Time Interaction analysis [16] showed that the change score is significantly greater for the active group relative to the change score for the control group (*p* = 0.0231, Cohen’s *d* = 0.56; Table 2 and Figure 2). Of the 25 MCI patients in the Placebo group, seven were below the cutoff score of MMSE (23/24) at follow-up, while in the Active group only two were (chi-square test, *p* = 0.0657). 

### 3.4. Analysis for Subgroups of Subjects with or without APOE4

The change of the MMSE score in the APOE4 (+) subgroup was −0.25 ± 1.4 for the Active subjects, and −1.6 ± 1.5 for the Placebo subjects (Treatment × Time Interaction, *p* = 0.0253, Cohen’s *d* = 0.9; Table 3). The analysis of the global CDR scores showed amelioration in the Active subjects, compared to the Placebo subjects (Treatment × Time Interaction, *p* = 0.0261, Cohen’s *d* = 1.19). In the APOE4 (-) subgroup, there was no significant difference between the two groups (Table 4).

### 3.5. The Concentration of Aβ1-42 in Plasma of APOE4 (+) Subjects

**Figure 3:**
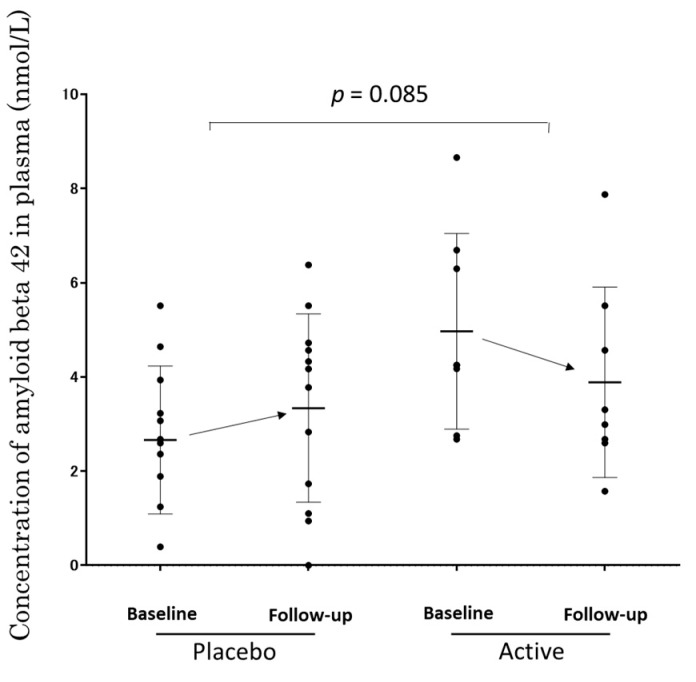
The concentration of Aβ1-42 in the plasma of the APOE4 (+) subjects measured by ELISA. A dot shows data from a subject. A column represents 25–75 percentile of the data. A bar shows the average of the data and ± SD.

Figure 3 shows the concentration of Aβ1-42 in the plasma measured by ELISA. The change of concentration during the trial was −1.08 ± 1.06 in the Active subjects (from 4.88 ± 2.08 to 3.79 ± 2.02), and 0.68 ± 3.04 in the Placebo subjects (from 2.57 ± 1.57 to 3.25 ± 2.0). A trend, but not significant, for the decrease in the concentration of amyloid beta 42 in the plasma was observed by oral ACS compared to a placebo (*p* = 0.085), implying an effect of the anserine and carnosine supplementation.

### 3.6. EEG Data

**Figure 4:**
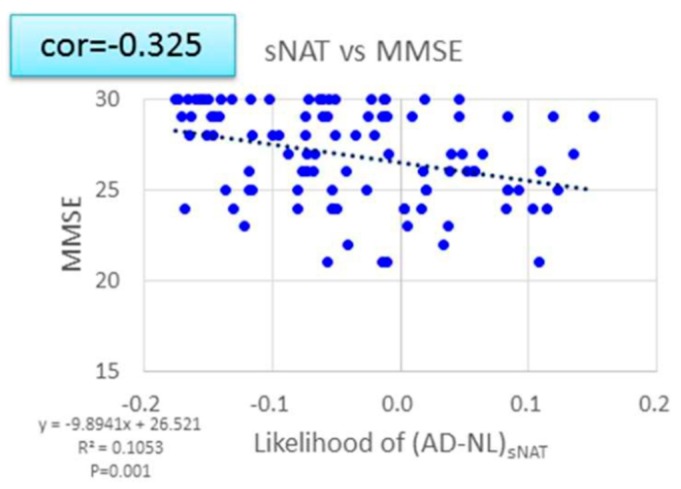
The correlation between the Neuronal Activity Topography (sNAT) score and Mini-Mental State Examination (MMSE) test score. sNAT scores were strongly correlated with the scores from MMSE (*p* < 0.001).

**Figure 5:**
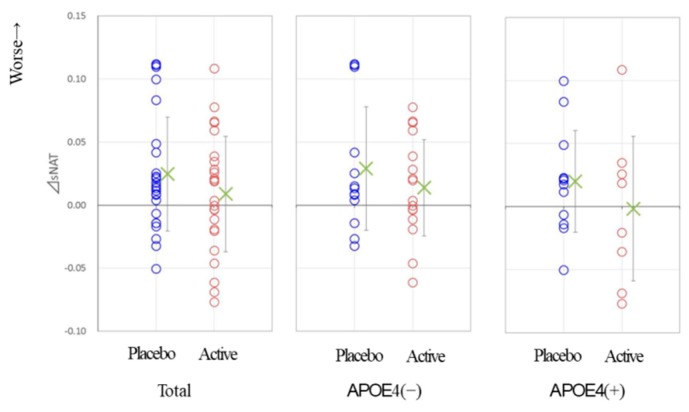
A tendency toward improved sNAT scores in APOE4 (+) MCI subjects after 12 weeks of ACS. P: Placebo group; A: Active group.

**Figure 6:**
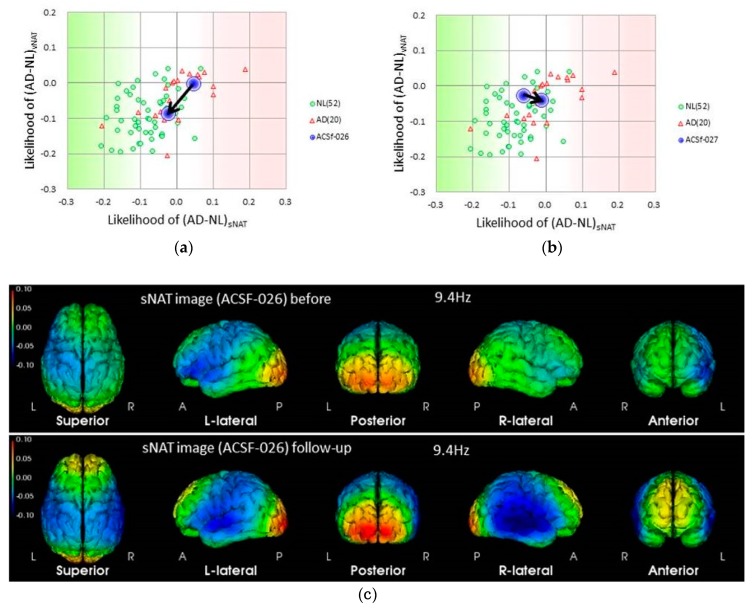
Electroencephalograms (EEG) based evaluation of the change of neural activity in APOE4/E4 MCI subjects by the supplementation. (**a**) Changes in the EEG pattern of subject #26 (Active group) from the start to the end of the trial, shown by blue circles in a two-dimensional NAT plot (sNAT compared to vNAT [23]) with data from both Alzheimer’s disease (AD) subjects (red triangles) and normal control subjects (normal (NL), green circles); (**b**) changes in the EEG pattern of subject #27 (Placebo group) from the start to the end of the trial; (**c**) sNAT images of subject #26 at the startup visit (baseline) and at follow-up. Note the elevated alpha-band activity at the occipital lobe and frontal lobes, suggesting improved EEG activity after ACS.

In this analysis, the cerebral neuronal activity is determined by the EEG spectrum intensity, sNAT (Neuronal Activity Topography) [23]. For the present report, we used databases collected in the Tone epidemiological investigation project, denoted as T-data [32], and showed the strong correlation between the sNAT value and the score of MMSE (Figure 4). Figure 5 shows that the deteriorated change in sNAT occurred in the placebo group but not in the active group. This tendency was robust in the APOE4(+) subjects. Figure 5 shows the two-dimensional visualization of sNAT and vNAT, another value from the EEG data analysis [23] for the change of the values from APOE4/4 MCI subjects. In this analysis, normal (NL) subjects (green dots) are mostly distributed on the left side of the histogram, and AD subjects (red triangles) are mostly distributed on the right (data for NL and AD subjects are from a previous study). The EEG pattern of an APOE4/4 patient in the Active group (subject #26) moved to the NL region after the 12-week trial period. In contrast, for an APOE4/4 patient in the Placebo group (subject #27) who was diagnosed as AD at follow-up, the EEG pattern had moved into the AD region. The APOE4/4 patient (subject #26) in the Active group was on a good course at the end of the trial (the score of gloCDR; from 0.5 to 0), consistent with the EEG analysis data (Figure 6). 

### 3.7. Clinical Safety

There were no reported or observed adverse events, severe or otherwise, during the trial. Data abnormalities, which were thought to be associated with the test supplement administration was not found in the blood analysis at follow-up.

## 4. Discussion

In this communication, we conducted a randomized, double-blind, placebo-controlled trial to investigate the effect of anserine and carnosine supplementation on MCI subjects. In the previous studies from our laboratory and others [16,17,18,19], it was shown that ACS helps preserve cognitive function in older adults with normal cognition and preserves brain blood flow especially in individuals carrying APOE4 [16,17,18,19]. Therefore, in the present study with subjects of MCI patients, we went on to an investigation of the cognitive function of APOE4 positive subjects, especially. Though statistically significant effects by ACS on the changes of cognitive tests during the trial were not obviously observed in the total subjects, the effect for the preservation of the cognitive function in APOE4 (+) MCI subjects may exist. It was shown in the difference of MMSE score changes from the baseline and to the follow-up between the two groups (from 27.1 to 26.9 in the Active and from 26.7 to 25.1 in the Placebo). In the CDR test, we detected a significant difference in the change of the gloCDR score between the two groups (*p* = 0.0261; −0.25 in the Active group and 0 in the Placebo) in APOE4 (+) subjects. EEG data, as well as *Aβ1-42* in the plasma data, supported the notion that ACS may help to maintain the cognitive function, especially in APOE4 (+) subjects.

In the total subjects, we detected a difference only in the change of the gloCDR score in total subjects between the two groups (Table 2). The transition from MCI to the normal cognitive status is called reversion, which is not rare without any specific medical intervention. The rate reported being about 24% in a meta-analysis [33]. CDR is a dementia rating instrument which requires information from a person closely observing the subject, and gloCDR has been often utilized to find reverters in patients, as a supplementary to clinical interview [26,27,33,34,35]. The score of gloCDR that corresponds to reversion is the change from 0.5 to 0. In the active group, the gloCDR score improved from 0.5 to 0 in half of the eight APOE4 (+) subjects, but in the placebo group only two of the 17 APOE4 (-) subjects did. The risk for dementia onset in reverters drops to about 40% of that in MCI individuals without reversion [34,35]. These observations might imply a possibility that ACS has a promotive effect for reversion, and, as a secondary effect, for the risk reduction of the AD onset, in APOE4 (+) MCI subjects.

The overall tendency in the number of diagnoses by clinical specialists in the study might be consistent with the potential of ACS for prevention from dementia onset. Of the 25 subjects, four in Placebo group developed dementia due to AD in the DSM-5, while all subjects in the Active group remained MCI at follow-up.

In this communication, the number of subjects suggested to become demented was different for each test since we reported independent test scores, though the efficacy of ACS might be shown in MMSE as well as in CDR. MMSE is a screening tool with good specificity for mild AD [36]. The fact that seven subjects in the Placebo group and only two subjects in the Active group went below the cut-point on MMSE might also reflect the efficacy of ACS for prevention from dementia. In CDR, a direct effect against developing dementia in MCI was not revealed clearly, compared to the assumable potential through promoting reversion from MCI to cognitive normal. One subject reached the gloCDR score of 1.0 or above (one ApoE4/E3 subject in the Placebo group with gloCDR 1.0 at follow-up, shown in Figure 3), and none in the Active group did, which was not significantly different. WMS-1 and WMS-2, and ADAS did not show cognitive amelioration by ACS in our study.

In our previous publication, we demonstrated cognitive amelioration by ACS on the delayed recall test WMS-2 with cognitive normal healthy elderly subjects [16,17]. The average WMS-2 score in the previous study was over 10 out of total 25, while that in the present study was around 5. ADAS is pointed out to be suited for use in mild-to-moderate AD dementia but lacks appropriate sensitivity of some items for MCI or mild dementia due to AD [34]. It may be supposed that the mismatching of test instruments and the subject population explain the failure to detect the effects of ACS in some tests in the present study. We experienced challenges in the selection of cognitive tests for assessment of cognitive function in the MCI stage and early dementia.

ACS might protect against the development of AD dementia and promote amelioration of cognitive decline in APOE4 (+) MCI individuals. We want to mention how ACS could help APOE4 (+) subjects. The EEG sNAT analysis showed a tendency toward improvement in the APOE4 (+) subgroup (Figure 5), in this EEG-based evaluation, locations with neuronal hypoactivity and undersynchrony in AD subjects are statistically correlated with the locations of regional cerebral blood flow (rCBF) reduction measured by SPECT [23,24,37]. In the previous study with cognitively healthy older adults [19], brain blood flow in the prefrontal area was preferentially preserved in APOE4 (+) individuals. In the present study, plasma amyloid beta 42 of APOE4 (+) MCI subjects showed a trend for decrease by ACS. It is reported that the plasma amyloid beta 42 is just within a moderate decrease during the preclinical or prodromal AD stages and in mild-to-moderate AD [38,39], and increased levels of amyloid beta is associated with vascular disease [38]. The observed trend in amyloid beta 42 concentration in plasma might reflect the beneficial effect of ACS on blood vessels when ACS ameliorated cognitive decline in APOE4 (+) MCI subjects.

In a transgenic AD mouse model, we have demonstrated that anserine supplementation, as well as carnosine supplementation, prevented memory deficits [40,41]; it also reversed the blood-vessel abnormality and elevated RAGE expression in these mice [40]. We have reported that anserine supplementation in AD model mice significantly prevented damage to brain microvascular pericytes, triggered by the accumulation of amyloid-beta peptides, toward the normal level [41]. In a series of elegantly coordinated preclinical and clinical studies, Zlokovic et al. demonstrated the role of brain blood-vessel pericytes in enabling brain microcapillaries to maintain the flow of brain blood and suggested that pericyte degeneration contributes to the etiology of AD carrying APOE4 [42,43,44,45]. In addition to the effect on pericytes, we have also reported that ACS suppressed the gene expression level of a blood chemokine, CCL24, in cognitively normal older adults [46]. It has been reported that APOE4 also has a considerable influence on peripheral blood macrophage, as well as brain microglia when these cells were treated with lipopolysaccharide (LPS). In these experiments, following LPS stimulation, apoE4-macrophage showed higher inflammatory reaction than apoE3-macrophage [47,48]. Therefore, it can be assumed that once anserine/carnosine supplements are ingested, these peptides directly and/or indirectly resulted in the protection of the degenerative cellular changes of brain microvascular pericytes which can be triggered by the accumulation of amyloid-beta peptides [8]; however, further studies are required to determine the precise mechanisms by which ACS affects brain function in APOE4 (+) individuals.

Our study has some limitations. First of all, the sample size adopted in the present study may not be sufficient to detect the score differences, if they were, in all the cognitive tests performed. Second, ACS was significantly affected in APOE4 (+) subjects, but not in subjects without the allele significantly. It can be possible that blood-vessel alterations occur more rapidly in ApoE4 (+) than in ApoE4 (-) individuals, as is implied in a previous study [19], but further study is necessary to verify the effect of anserine and carnosine in MCI subjects. We are underway to prepare and start the next RCT study to evaluate the effect of ACS. Third, changes in EEG did not differ between the two groups statistically. It may be due to the small sample size. Further study with appropriate sample size and follow-up duration would help us understand the significance of these results for various populations. Authors should discuss the results and how they can be interpreted in perspective of previous studies and the working hypotheses. The findings and their implications should be discussed in the broadest context possible. Future research directions may also be highlighted.

## 5. Conclusions

This study is the first RCT concerning the effects of ACS in MCI subjects. The results did not indicate that ACS has significant efficacy on cognitive change of MCI subjects but might suggest that beneficial effect in APOE4(+) MCI subjects exists, and thereby, ACS might help to prevent a transition from MCI to AD as a secondary effect in APOE4 (+) MCI subjects. Furthermore, changes in sNAT scores, which are based on the EEG analysis, may be a useful biomarker for assessing these benefits.

## Figures and Tables

**Table 1:** Subject characteristics ^a^.

	Active Group	Placebo Group	*p* Value
Age	72.9 ± 8.8 ^b^	73.6 ± 6.1	0.75 ^c^
Gender (M/F)	12/13	12/13	
BMI	22.2 ± 2.8	21.5 ± 2.6	0.46 ^c^
Years of education	13.9 ± 2.5	14.1 ± 2.8	0.83 ^c^
APOE4 positive/negative	8/17	12/13	0.25 ^d^

^a^ Data are shown for 50 subjects who completed the trial and follow-up tests. ^b^ Mean ± Standard Deviation. ^c^ *p* value was determined by the student’s t-test. ^d^ *p* value was by the chi-square test.

**Table 2:** Neuropsychological test scores for all subjects ^a^.

	Startup		Follow-Up		Treatment × Time Interaction ^b^		
	Active	Placebo	Active	Placebo	Active	Placebo	*p* Value
MMSE	27.5 ± 2.2	27.0 ± 2.1	27.1 ± 2.5	26.0 ± 3.4	−0.4 ± 1.6	−1.0 ± 1.8	0.252
gloCDR	0.5	0.5	0.38 ± 0.2	0.48 ± 0.2	−0.12 ± 0.22	−0.02 ± 0.18	0.0231
CDRsob	0.82 ± 0.45	1.04 ± 0.53	0.80 ± 0.5	1.12 ± 0.77	−0.02 ± 0.51	0.12 ± 0.79	0.583
WMS-1	7.8 ± 4.4	7.0 ± 4.5	9.1 ± 4.4	8.2 ± 5.7	1.2 ± 2.8	1.2 ± 3.1	0.591
WMS-2	5.3 ± 4.4	5.6 ± 4.6	6.7 ± 5.2	6.4 ± 6.1	1.4 ± 3.0	0.7 ± 3.0	0.975
ADAS	12.3 ± 7.4	15.5 ± 7.6	13.0 ± 8.5	15.4 ± 8.6	0.8 ± 4.4	−0.2 ± 4.7	0.320
GDS	2.5 ± 1.9	2.5 ± 2.4	2.6 ± 2.9	2.6 ± 2.1	0.1 ± 2.7	0.1 ± 2.0	0.943

^a^ Data are shown in mean ± Standard Deviation. ^b^ The change between start-up and follow-up.

**Table 3:** Clinical Dementia Rating (CDR) and psychological test scores for apolipoprotein E ε4 allele (APOE4) (+) subjects ^a^.

	Startup		Follow-Up		Treatment × Time Interaction ^b^		
	Active	Placebo	Active	Placebo	Active	Placebo	*p* Value
MMSE	27.1 ± 2.4	26.7 ± 2.3	26.9 ± 2.3	25.1 ± 3.6	−0.25 ± 1.4	−1.6 ± 1.5	0.0253
gloCDR	0.5	0.5	0.25 ± 0.27	0.50 ± 0.21	−0.25 ± 0.27	0 ± 0.21	0.0261
CDRsob	0.69 ± 0.37	1.25 ± 0.50	0.75 ± 0.65	1.33 ± 0.86	0.06 ± 0.68	0.08 ± 0.82	0.406
WMS-1	9.3 ± 3.4	6.5 ± 4.9	8.4 ± 3.1	6.8 ± 5.2	−0.88 ± 2.9	0.25 ± 2.5	0.592
WMS-2	5.8 ± 3.7	4.9 ± 4.5	6.4 ± 4.4	4.6 ± 5.5	0.63 ± 3.2	−0.33 ± 2.4	0.670
ADAS	10.9 ± 5.8	17.6 ± 8.9	12.2 ± 9.4	17.5 ± 9.9	1.3 ± 6.4	−0.13 ± 5.3	0.207
GDS	1.6 ± 1.1	2.1 ± 1.4	1.4 ± 2.0	2.6 ± 2.2	−0.25 ± 2.7	0.5 ± 1.9	0.165
GDS	2.5 ± 1.9	2.5 ± 2.4	2.6 ± 2.9	2.6 ± 2.1	0.1 ± 2.7	0.1 ± 2.0	0.943

^a^ We performed the same statistical analysis, described in Table 2. ^b^ The change between start-up and follow-up.

**Table 4:** CDR and psychological test scores for APOE4 (−) subjects ^a^.

	Startup		Follow-Up		Treatment × Time Interaction ^b^		
	Active	Placebo	Active	Placebo	Active	Placebo	*p* Value
MMSE	27.6 ± 2.1	27.3 ± 2.0	27.2 ± 2.6	26.8 ± 3.1	−0.41 ± 1.7	−0.46 ± 1.9	0.840
gloCDR	0.5	0.5	0.44 ± 0.17	0.46 ± 0.14	−0.059 ± 0.17	−0.038 ± 0.14	0.477
CDRsob	0.88 ± 0.49	0.85 ± 0.47	0.82 ± 0.43	0.92 ± 0.64	−0.059 ± 0.43	0.077 ± 0.76	0.602
WMS-1	7.2 ± 4.8	7.5 ± 4.2	9.4 ± 4.9	9.5 ± 6.1	2.2 ± 2.3	2.0 ± 3.5	0.993
WMS-2	5.1 ± 4.8	6.3 ± 4.6	6.8 ± 5.6	8.0 ± 6.5	1.7 ± 2.9	1.7 ± 3.3	0.997
ADAS	12.9 ± 8.2	13.6 ± 5.9	13.5 ± 8.2	13.4 ± 7.1	0.54 ± 3.3	−0.20 ± 4.3	0.697
GDS	2.9 ± 2.0	2.8 ± 3.1	3.2 ± 3.1	2.5 ± 2.1	0.29 ± 2.8	−0.30 ± 2.0	0.676

^a^ We performed the same statistical analysis, described in Table 2. ^b^ The change between start-up and follow-up.

## Supplementary Materials

The following are available online at https://www.mdpi.com/2072-6643/11/7/1626/s1, Table S1: Data from blood tests, Table S2. Estimated anserine/carnosine daily intake from meals.
