# Supplementary material for: Effects of Anserine/Carnosine Supplementation on Mild Cognitive Impairment with APOE4
Source: Nutrients. 2019 Jul 17;11(7):1626. doi: 10.3390/nu11071626 (PMC6683059; doi:10.3390/nu11071626)
Supplement: Supplementary file 1 [file nutrients-11-01626-s001.pdf]

Table S1. Data from blood tests [a.]

	Test	Unit	Active Group Ave.± SEM	Placebo Group Ave.± SEM	*p* value
Baseline.	AST.	(μ/L).	22.4±1.9.	23.4±1.4.	0.68..
	ALT	(μ/L)	18.3±1.5	16.4±1.2	0.31
	ALP	(μ/L)	215.9±32.2	188.6±10.7	0.42
	LD	(μ/L)	195.8±7	192.7±5	0.72
	γ-GT	(μ/L)	30.8±6	26.8±3.1	0.56
	Cholinesterase	(μ/L)	327.6±16	300.2±13.5	0.2
	Creatine Kinase	(μ/L)	96.2±8	124.2±20.2	0.2
	Creatinine	(mg/dL)	0.7±0	0.7±0	0.61
	Blood Urea Nitrogen	(mg/dL)	18.8±0.9	16.2±1	0.07
	Uric Acid	(mg/dL)	4.8±0.3	4.4±0.2	0.33
	Total Cholesterol	(mg/dL)	216.6±7.4	209.2±6.9	0.47
	Triglyceride	(mg/dL)	115.9±15.4	94.6±9.8	0.25
	Serum Amylase	(μ/L)	75.8±5.2	98±6.3	0.01
	Fasting glucose	(mg/dL)	104±3.3	97.1±1.8	0.07
	HDL cholesterol	(mg/dL)	69±4	76.4±4.1	0.2
	LDL cholesterol	(mg/dL)	124.6±6.3	112.8±5.1	0.15
	White Blood Cell	(/μL)	5504±289	5572±227.6	0.85
	Red Blood Cell	($\times 10^4$/μL)	448±9	433.1±9.3	0.26
	Hemoglobin	(g/dL)	14±0.2	13.7±0.3	0.5
	Hematocrit	(%)	42.7±0.7	41.7±0.8	0.32
	MCV	(fL)	95.6±0.8	96.4±1.2	0.61
	MCH	(pg)	31.2±0.3	31.8±0.5	0.35
	MCHC	(g/dL)	32.7±0.2	32.9±0.2	0.48
	Platelet	($\times 10^4$/μL)	22.4±1	22.2±1.1	0.88
Followup	AST	(μ/L)	22.5±2.2	23.9±1.2	0.6

	ALT	(μ/L)	18.7±2.2	16.7±1	0.42
	ALP	(μ/L)	231.8±43.7	192.8±11	0.39
	LD	(μ/L)	200.2±6.6	202.4±6	0.8
	γ-GT	(μ/L)	35.7±9.1	28.2±3.2	0.44
	Cholinesterase	(μ/L)	321.8±16.6	299.5±12.4	0.29
	Creatine Kinase	(μ/L)	95.4±8.6	129.1±17.7	0.09
	Creatinine	(mg/dL)	0.8±0	0.7±0	0.82
	Blood Urea Nitrogen	(mg/dL)	17.6±1	16.9±1	0.6
	Uric Acid	(mg/dL)	4.8±0.2	4.8±0.2	0.86
	Total Cholesterol	(mg/dL)	217.2±7.6	215.1±7.4	0.84
	Triglyceride	(mg/dL)	131.7±13.7	104.7±13.6	0.17
	Serum Amylase	(μ/L)	82.4±7.3	96.6±6	0.14
	Fasting glucose	(mg/dL)	122.1±8.7	101.2±3.4	0.03
	HDL cholesterol	(mg/dL)	69±4	77.9±4.8	0.16
	LDL cholesterol	(mg/dL)	122.4±4.9	113.9±5.7	0.26
	White Blood Cell	(/μL)	5584±290.3	5320±247.2	0.49
	Red Blood Cell	($\times 10^4$/μL)	447.3±8.7	437.4±8.5	0.42
	Hemoglobin	(g/dL)	13.9±0.2	13.8±0.2	0.72
	Hematocrit	(%)	43.1±0.7	42.3±0.7	0.42
	MCV	(fL)	96.5±0.8	97±1.2	0.72
	MCH	(pg)	31.2±0.3	31.6±0.5	0.4
	MCHC	(g/dL)	32.3±0.2	32.6±0.2	0.32
	Platelet	($\times 10^4$/μL)	21.6±0.9	21.2±0.8	0.73
Followup - Baseline	AST	(μ/L)	0.1±0.7	0.5±1	0.75
	ALT	(μ/L)	0.4±1.3	0.4±0.9	0.98
	ALP	(μ/L)	15.9±12.3	4.3±5.8	0.4
	LD	(μ/L)	4.4±2.5	9.7±4.4	0.3
	γ-GT	(μ/L)	4.9±3.5	1.3±1.4	0.35
	Cholinesterase	(μ/L)	-5.8±4.4	-0.7±4.4	0.42
	Creatine Kinase	(μ/L)	-0.7±5.3	4.9±12.1	0.67
	Creatinine	(mg/dL)	0±0	0±0	0.19

Blood Urea Nitrogen	(mg/dL)	-1.2±1	0.6±0.9	0.17
Uric Acid	(mg/dL)	0±0.1	0.4±0.1	0.01
Total Cholesterol	(mg/dL)	0.6±6	5.9±3.7	0.45
Triglyceride	(mg/dL)	15.8±8.7	10.1±8	0.64
Serum Amylase	(μ/L)	6.6±4.4	-1.4±2	0.1
Fasting glucose	(mg/dL)	18.2±8.8	4.1±3.2	0.14
HDL cholesterol	(mg/dL)	0±1.5	1.5±2.5	0.62
LDL cholesterol	(mg/dL)	-2.2±5.4	1.1±3.1	0.59
White Blood Cell	(/μL)	80±113.9	-252±228	0.2
Red Blood Cell	($\times10^4$/μL)	-0.7±5.6	4.3±3.8	0.47
Hemoglobin	(g/dL)	-0.1±0.1	0.1±0.1	0.49
Hematocrit	(%)	0.4±0.5	0.6±0.4	0.66
MCV	(fL)	0.9±0.4	0.7±0.4	0.72
MCH	(pg)	-0.1±0.2	-0.1±0.1	0.72
MCHC	(g/dL)	-0.4±0.2	-0.3±0.2	0.73
Platelet	($\times10^4$/μL)	-0.8±0.6	-1±0.5	0.8

[a] *p* value was determined by student's t-test.

Table S2. Estimated anserine/carnosine daily intake from meals [a].

	**Food**	**Active Group Ave. ± SEM**	**Placebo Group Ave. ± SEM**	***p* value**
Anserine (mg/day)	Poultry	169.8±29	174.3±25.2	0.91
	Pork	5.2±0.8	6.2±0.9	0.42
	Beef	4.9±0.9	4.7±1.3	0.92
	Red meat Fish	19.4±2.4	18.5±2.8	0.8
	Blue back Fish	0.1±0	0±0	0.16
	White Fish	0.3±0	0.4±0.1	0.34
	Salmon	106.2±17.8	143.1±22.9	0.21
	Eel	0±0	0±0	N.D.
	Anserine (Total)	305.9±35	347.3±34.6	0.4

Carnosine (mg/day)	Poultry	47.6±8.1	48.9±7.1	0.91
	Pork	69.7±10.8	82.7±11.7	0.42
	Beef	23.6±4.2	22.9±6.2	0.92
	Red meat Fish	15.4±1.9	14.7±2.2	0.8
	Blue back Fish	7.8±0.9	6.1±0.9	0.16
	White Fish	0±0	0±0	N.D.
	Salmon	0±0	0±0	N.D.
	Eel	13.8±5	7.6±1.2	0.24
	Carnosine (Total)	177.9±18.6	182.9±21.2	0.86

[a] *p* value was determined by student's t-test.
